# Supplementary material for: Modelling Skylarks (Alauda arvensis) to Predict Impacts of Changes in Land Management and Policy: Development and Testing of an Agent-Based Model
Source: PLoS One. 2013 Jun 6;8(6):e65803. doi: 10.1371/journal.pone.0065803 (PMC3675089; doi:10.1371/journal.pone.0065803)
Supplement: Supporting Information S4 — The skylark ODdox as a zipped archive. (ZIP) [file pone.0065803.s004.zip › Skylark_ODdox/classes.html]

ALMaSS Skylark ODdox: Class Index


ALMaSS Skylark ODdox
 2.0


- Main Page
- Related Pages
- Classes
- Files

- Class List
- Class Index
- Class Hierarchy
- Class Members

Class Index

A | B | C | D | E | F | G | H | I | L | M | N | O | P | R | S | T | U | V | W | Y

|  |  |  |  |  |
| --- | --- | --- | --- | --- |
| A | Diffusor | Nestling\_struct | RuralResidential | UserDefinedFarm13 |
|  | E | NonVegElement | S | UserDefinedFarm14 |
| ActivePit |  | NoPesticideBaseFarm |  | UserDefinedFarm15 |
| aDate | EdgeGrowingPoints | NoPesticideNoPFarm | Saltwater | UserDefinedFarm16 |
| AdultSkylark\_struct | Edges | O | SandDune | UserDefinedFarm17 |
| AgroChemIndustryCerealFarm1 | EGP\_Data |  | ScalablePositionMap | UserDefinedFarm18 |
| AgroChemIndustryCerealFarm2 | F | Orchard | Scrub | UserDefinedFarm19 |
| AgroChemIndustryCerealFarm3 |  | OrchardBand | SimplePositionMap | UserDefinedFarm2 |
| AmenityGrass | Farm | OrchardGrass | skClutch\_struct | UserDefinedFarm20 |
| AnimalPosition | FarmEvent | OrganicCattle | SkQualGrid | UserDefinedFarm21 |
| APoint | FarmManager | OrganicPig | skSpiralResult | UserDefinedFarm22 |
| B | Field | OrganicPlant | SkTerritories | UserDefinedFarm23 |
|  | FieldBoundary | P | skTerritory\_struct | UserDefinedFarm24 |
| BaitLocation | ForestElement |  | skTTerritory | UserDefinedFarm25 |
| BareRock | Freshwater | Parkland | Skylark\_Adult | UserDefinedFarm26 |
| BeetleBank | G | PermanentSetaside | Skylark\_Base | UserDefinedFarm27 |
| BinaryMapBase |  | PermCropData | Skylark\_Clutch | UserDefinedFarm28 |
| Building | Garden | PermPasture | Skylark\_Female | UserDefinedFarm29 |
| BuiltUpWithParkland | GreenElement | PermPastureLowYield | Skylark\_Male | UserDefinedFarm3 |
| C | H | PermPastureTussocky | Skylark\_Nestling | UserDefinedFarm30 |
|  |  | Pesticide | Skylark\_Population\_Manager | UserDefinedFarm31 |
| Calendar | Heath | PesticideEvent | Skylark\_PreFledgeling | UserDefinedFarm32 |
| CfgBase | HedgeBank | PesticideTrialControl | Skylark\_struct | UserDefinedFarm33 |
| CfgBool | Hedges | PesticideTrialToxicControl | SmallRoad | UserDefinedFarm34 |
| CfgFloat | I | PesticideTrialTreatment | Starter | UserDefinedFarm35 |
| CfgInt |  | PitDisused | StoneWall | UserDefinedFarm36 |
| CfgStr | IDMap | Point | Suburban | UserDefinedFarm4 |
| Coast | IntArray100 | PolygonDataVector | T | UserDefinedFarm5 |
| CompareFarmNum | L | Population\_Manager |  | UserDefinedFarm6 |
| CompareState |  | PositionMap | TALMaSSObject | UserDefinedFarm7 |
| CompareStateR | Landscape | PreFledgeling\_struct | TAnimal | UserDefinedFarm8 |
| CompareX | LargeRoad | priority\_queue | tpct | UserDefinedFarm9 |
| CompareY | LE | probe\_data | Track | V |
| Configurator | LE\_TypeClass | R | TSkylarkList |  |
| ConiferousForest | LowPriority |  | U | vector |
| ConventionalCattle | LowPriPair | Railway |  | VegElement |
| ConventionalPig | M | RasterMap | UnsprayedFieldMargin | W |
| ConventionalPlant |  | rectangle | Urban |  |
| ConvMarginalJord | MapErrorMsg | River | UrbanNoVeg | Weather |
| Copse | Marsh | RiversidePlants | UrbanPark | Y |
| Crop | MixedForest | RiversideTrees | UserDefinedFarm |  |
| CropData | MovementMap | RoadsideVerge | UserDefinedFarm1 | YoungForest |
| CropGrowth | MovementMap16 | RodenticideManager | UserDefinedFarm10 |  |
| CropRotation | N | RoeDeerInfo | UserDefinedFarm11 |  |
| D |  | Rotation | UserDefinedFarm12 |  |
|  | NaturalGrass |  |  |  |
| DeciduousForest |  |  |  |  |
|  |  |  |  |  |

A | B | C | D | E | F | G | H | I | L | M | N | O | P | R | S | T | U | V | W | Y


- Generated on Thu Jan 10 2013 13:15:39 for ALMaSS Skylark ODdox by
   1.8.1.1
